# Left ventricular longitudinal function is reduced but partially compensated by increased radial function after heart transplantation

**DOI:** 10.1016/j.jhlto.2025.100308

**Published:** 2025-05-31

**Authors:** Grunde Gjesdal, Anna Székely, Henrik Engblom, Håkan Arheden, Oscar Ö Braun, Katarina Steding-Ehrenborg

**Affiliations:** a Department of Cardiology, Clinical Sciences, Lund University and Skåne University Hospital, Lund, Sweden; b Clinical Physiology, Department of Clinical Sciences Lund, Lund University, Lund, Sweden; c Department of Clinical Physiology, Skåne University Hospital, Lund, Sweden

**Keywords:** Heart transplant, Cardiac magnetic resonance imaging, Left ventricular atrioventricular plane displacement, Longitudinal heart function, Longitudinal contribution to stroke volume

## Abstract

**Background:**

In healthy hearts, left ventricular atrioventricular plane displacement (LVAVPD) measured by cardiac magnetic resonance (CMR) contributes to ∼60% of stroke volume. LVAVPD has been shown to correlate with maximal cardiac output and exercise capacity and is an independent predictor of outcomes in patients with heart failure. We aimed to assess if longitudinal pumping is altered, if LVAVPD is associated with exercise capacity, and if any difference in longitudinal pumping could be explained by the presence of a right bundle branch block (RBBB) in heart-transplanted patients.

**Method:**

This single-center study included 34 heart-transplanted patients who had undergone CMR and a cardiopulmonary exercise test as part of a clinical post-transplant surveillance program. Data was compared to 34 healthy sex- and age-matched controls.

**Results:**

Heart-transplanted patients had decreased LVAVPD (10.3 vs 13.7 mm, *p* < 0.01), lower longitudinal contribution (46% vs 53%, *p* < 0.01), and lower septal contribution (−3% vs 8%, *p* < 0.01) to stroke volume compared to controls. Furthermore, the lateral contribution was increased (44% vs 28%, *p* < 0.01) in the heart-transplanted patients. Longitudinal contribution to stroke volume was neither associated with exercise capacity (*p* = 0.20) nor cardiac output at rest (*p* = 0.62). There was no difference in LVAVPD in patients with and without RBBB (*p* = 0.81).

**Conclusion:**

Heart-transplanted patients have decreased left ventricular longitudinal function compared to healthy controls, in part compensated by an augmented lateral function. Longitudinal function is not associated with cardiac output at rest or exercise capacity in this patient group. Whether the altered pumping mechanics seen are associated with outcome remains to be investigated.

## Background

### Left ventricular atrioventricular plane displacement (LVAVPD)

In healthy hearts, LVAVPD measured by cardiac magnetic resonance (CMR) at rest is correlated with both maximal cardiac output and exercise capacity.^1^ It is also an independent predictor of outcomes in patients, such as death and lung transplantation in pulmonary arterial hypertension^2^ and death in patients with heart failure with reduced ejection fraction.^3^ It has previously^4^ been shown numerically close to the echocardiographic measure mitral annular plane systolic excursion (MAPSE), although it should be noted that they are not equal, as MAPSE also includes the effect of radial movement.

The LVAVPD, and thus the longitudinal pumping of the heart, is the main contributor to ventricular stroke volume, contributing with approximately 60% of the total stroke volume from the left ventricle. The remaining 40% results from radial pumping.^5^, ^6^ From an energy-conserving perspective, longitudinal pumping with no or minor outer volume variation is optimal.^7^ Whether longitudinal function assessed by LVAVPD is reduced and if the associations with cardiac output, exercise capacity, and outcome are maintained in heart transplant recipients have not been previously investigated.

### Heart transplantation and right bundle branch block (RBBB)

RBBB is present in up to 50% of heart transplant recipients.^8^, ^9^ Sandhu et al showed no hemodynamic effects of RBBB measured by right heart catheterization.^8^ However, newly developed or progressive RBBB in transplanted recipients has been associated with decreased left ventricular ejection fraction (LVEF).^10^ As RBBB affects ventricular function, it may also affect longitudinal pumping.

### Study aim

The aims of this study were to 1) determine LVAVPD and assess if longitudinal and radial contributions to stroke volume differed in heart transplant recipients compared to a group of matched healthy controls, 2) to investigate if there was an association between LVAVPD and cardiac output at rest, or between LVAVPD and maximal exercise capacity as measured by cardiopulmonary exercise testing (CPET), and 3) to assess if there was a difference in the regional contribution to stroke volume in patients with or without a RBBB.

## Methods

### Study population and study design

Thirty-four participants (mean age 51 years [95% CI 46-56 years], 11 [32%] female) in the clinical follow-up program at the heart transplant unit at Skåne University Hospital, Lund, Sweden, were retrospectively included in the analysis. All patients had undergone CMR and a CPET as part of their routine post-transplant surveillance program between November 2019 and October 2022.

Thirty-four age- and sex-matched healthy controls were retrospectively included from previous studies. Controls were volunteers, self-assessed as healthy, non-smokers without daily medication. All the controls had normal blood pressure, resting electrocardiogram (ECG), and CMR at inclusion.

CPET investigations were not available from the control cohort. Data on time from transplant, prior or current allograft rejection, presence of macroscopic cardiac allograft vasculopathy (CAV) measured by computed tomography or angiography or by intravascular ultrasound (IVUS), immunosuppressive medication, other relevant daily medication, blood pressure, and the presence or absence of RBBB at ECG at the time of CMR were collected from the patient’s medical records.

The study was approved by the regional ethics committee in Lund, Sweden (application 2004/741 with addendum 2018/948 and application 2013/319) and by the Swedish Ethical Review Authority (Dnr 2013/900, 2015/248, and 2018/431) and conducted in accordance with the International Society for Heart and Lung Transplantation’s Ethics statement. All participants provided written informed consent.

### CMR acquisition and analysis

Cardiac MR was performed using Siemens Magnetom Aera 1.5 T and Siemens Magnetom Sola 1.5 T scanners (Siemens Healthcare, GmbH, Erlangen, Germany). Localizers and scout images were acquired to define left ventricular short-axis and long-axis planes (2-, 3- and 4-chamber views). Short-axis images were delineated according to the Society for Cardiovascular Magnetic Resonance Board of Trustees Task Force protocol to obtain left ventricular mass, volume, stroke volume, and ejection fraction. Trabeculations and papillary muscles were included in the intracavitary volume.^11^

**Figure 1:**
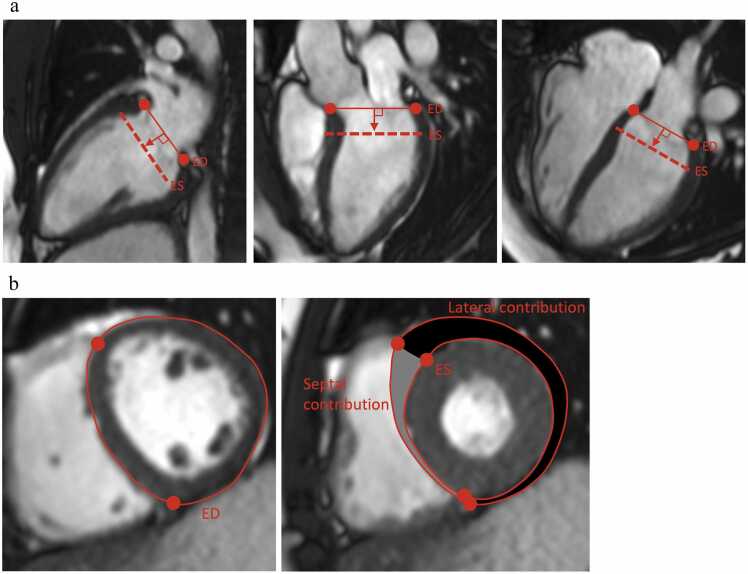
**a.** Atrioventricular plane definition. At end-diastole, six markers were placed at the atrioventricular junction in the 2-, 3-, and 4-chamber views (red dots). Left ventricular atrioventricular plane displacement was defined as the average movement of the six markings toward the apex. **b.** Septal and lateral contribution to stroke volume. The septal part of the left ventricle was defined as the part between the anterior and inferior insertion of the right ventricle, whereas the lateral part is defined by the remaining free wall. Septal and lateral contribution to stroke volume was calculated by the milliliter of blood displaced by the septal and lateral movement during systole divided by the estimated stroke volume.

Calculation of LVAVPD was performed using Segment v3.3 software (https://medviso.com/segment/) according to the methodology previously described by Carlsson and colleagues,^5^, ^12^ and validated by Seemann et al^13^ The atrioventricular plane was defined by manually marking the inferoseptal and anterolateral mitral annular hinge in the 4-chamber view, the anterior and inferior points in the 2-chamber view, and the inferolateral and anteroseptal points in the 3-chamber view. Tracking was manually adjusted in end-diastole (ED) and end-systole (ES) when needed. Left ventricular AVPD was calculated as the perpendicular mean shortening distance in millimeters between six markings indicating the atrioventricular plane and the apex (Figure 1a).

Longitudinal contribution to stroke volume (SV_long%_) as well as radial contribution divided into septal and lateral contribution was calculated using methodology previously described.^5^, ^6^ SV_long%_ was determined by multiplying the LVAVPD with the largest epicardial short-axis area of the left ventricle. Septal contribution to stroke volume (SV_sept%_) was calculated from the difference between the ED and ES area enclosed by the septal epicardial contours and the insertion of the right ventricle in the short-axis images. Lateral contribution (SV_lat%_) was similarly calculated from the difference between the ED and ES area enclosed by the lateral LV epicardial contours and the right ventricular insertion points in the short-axis images (Figure 1b).

### CPET

Peak oxygen uptake measured in ml x kg^−1^ x min^−1^ and respiratory exchange ratio (RER) were assessed from CPET examinations according to accredited clinical routines and was performed as part of the routine post-transplant surveillance program. Protocols were individually adapted based on age, sex, and physical activity level and chosen to yield an exercise duration of 8-12 min.^14^

### Statistical analysis

Statistical analysis was performed using the software Graph Pad Prism version 9.5.1 (GraphPad Software Inc., La Jolla, CA, USA). Normal distribution was tested visually and by D′Agostino Pearson’s test. Mean values were compared using a two-sided unpaired *t*-test. Univariate linear regression was used to analyze potential associations between LVAVPD and transplant status, heart rate (HR), systolic blood pressure, left ventricular volume, stroke volume, body surface area (BSA), sex, and age. Multiple linear regression models were used to assess the association between factors that were significantly associated in the univariate linear analysis. Results are presented as mean with 95% confidence intervals (95% CI) for normal distributed data and as median and interquartile range for non-parametric data. A *p*-value below 0.05 was considered statistically significant.

## Results

### Patient characteristics

Patient and healthy control characteristics are outlined in Table 1. There was no difference in sex, age, or BSA between the heart-transplanted patients and controls.

**Table 1:** Patient Characteristics

	Heart-transplanted patients (*n* = 34)	Healthy controls (*n* = 34)	*P*-value
Female sex	11 (32%)	11 (32%)	1.00
Age, years	51 (46-56)	50 (46-55)	0.88
BSA, m^2^	2.0 (1.9-2.0)	1.9 (1.9-2.0)	0.59
Year after transplant	5 (3-7)	n/a	
ECG with RBBB-pattern	11 (32%)	n/a	
Prior or current rejection		n/a	
Prior ACR ≥ 2R	3 (10%)		
Prior AMR	1 (3%)		
Current ACR ≥ 2R	0		
Current AMR	0		
CAV according to coronary CT or angiogram	2 (6%)		
CAV according to IVUS	11 (32%)		
Immunosuppression	34 (100%)	n/a	
Prednisone	24 (70%)		
Tacrolimus	28 (82%)		
Ciclosporin	2 (6%)		
Everolimus	7 (21%)		
Mycophenolate mofetil	30 (88%)		
Azathioprine	2 (6%)		
Other daily medication		n/a	
Acetylsalicylic acid	27 (79%)		
Betablockers	6 (18%)		
Calcium channel blockers	18 (53%)		
Ivabradine	0		
ACE-inhibitors/Angiotensin receptor blockers	18 (53%)		
Mineralocorticoid receptor antagonists	4 (12%)		
Loop-diuretics	5 (15%)		
Thiazides	0		
Statins	33 (97%)		
Insulin or other diabetes medication	8 (24%)		

Categorical data are presented as numbers with percentage, and continuous data are presented as mean with 95% CI.

There was no statistically significant difference in sex measured by Fishers exact test, or in age or body size measured by unpaired *T*-test.

Abbreviations: ACE, angiotensin converting enzyme; ACR, Acute cellular rejection; AMR, Antibody-mediated rejection; BSA, body surface area; CAV, cardiac allograft vasculopathy; CT, computed tomography; ECG, electrocardiogram; IVUS, intravascular ultrasound; n/a, not applicable; RBBB, right bundle branch block.

None of the 34 heart-transplanted patients were suspected or assessed to have ongoing acute rejection at the time of investigation. Three (8.8%) patients had previously received treatment for acute cellular rejection (ACR), and one had previously been diagnosed with antibody-mediated rejection (AMR). One of the three patients with previous ACR 2R (2.9%) was diagnosed more than 6 months before the CMR investigation. For the remaining two, as well as the patient with previous AMR, more than 2 years had passed since rejection was suspected. One of the three patients (2.9%) with ACR had a decreased LVEF at the time that rejection was diagnosed, but the ejection fraction normalized shortly after rejection treatment.

All patients were evaluated with coronary angiogram or computed tomography angiogram for assessment of CAV within one year after the CMR investigation. Mild CAV (CAV_1_) was present in two patients (5.9%), while the rest had no signs of CAV. All heart-transplanted patients received ≥2 oral immunosuppressive drugs as well as other transplant-related and hemodynamically active medication, with distribution between medications outlined in Table 1.

### Cardiac mass, volume, and function

There was no difference in left ventricular mass, left ventricular end-diastolic volumes, or ejection fraction between transplanted patients and healthy controls. However, left ventricular end-diastolic volumes indexed to BSA were slightly smaller in transplanted patients compared to healthy controls (Table 2).

**Table 2:** CMR Results

Analyzed variable	Heart-transplanted patients (*n* = 34)	Healthy controls (*n* = 34)	*P*-value
LVM (g)	104 (98-111)	99 (92-105)	0.22
LVMI (g/m^2^)	53 (50-55)	51 (48-54)	0.30
LVEDV (ml)	158 (148-169)	172 (163-181)	0.05
LVEDVI (ml/m^2^)	80 (76-85)	89 (84-93)	0.01
LVSV (ml)	90 (83-96)	100 (95-106)	0.01
LVSVI (ml/m^2^)	46 (43-49)	52 (49-54)	<0.01
LVEF (%)	57 (55-59)	59 (57-61)	0.16
HF (beats/min)	81 (77-85)	64 (61-67)	<0.01
SBP (mmHg)	123 (118-129)	124 (120-129)	0.79
CO (L/min)	7.2 (6.7-7.7)	6.4 (6.1-6.7)	0.01
CI (L/min/m^2^)	3.7 (3.4-3.9)	3.3 (3.2-3.4)	0.01
LVAVPD (mm)	10.3 (9.8-10.8)	13.7 (13.0-14.4)	<0.01
Longitudinal contribution to LVSV (%)	46 (44-48)	53 (50-56)	<0.01
Septal contribution to LVSV (%)	−3 (−5-(−1))	8 (7-10)	<0.01
Lateral contribution to LVSV (%)	44 (40-48)	28 (24-31)	<0.01

Data presented as mean with (95% CI). Difference between means analyzed by an unpaired *T*-test.

Abbreviations: CI, cardiac index; CO, cardiac output; HF, heart frequency; LVAVPD, left ventricular atrioventricular plane displacement; LVEDV, left ventricular end-diastolic volume; LVEDVI, left ventricular end-diastolic volume index; LVEF, left ventricular ejection fraction; LVM, left ventricular mass; LVMI, left ventricular mass index; LVSV, left ventricular stroke volume; LVSVI, left ventricular stroke volume index; SBP, Systolic blood pressure.

### Longitudinal and regional contribution to stroke volume and cardiac output

**Figure 2:**
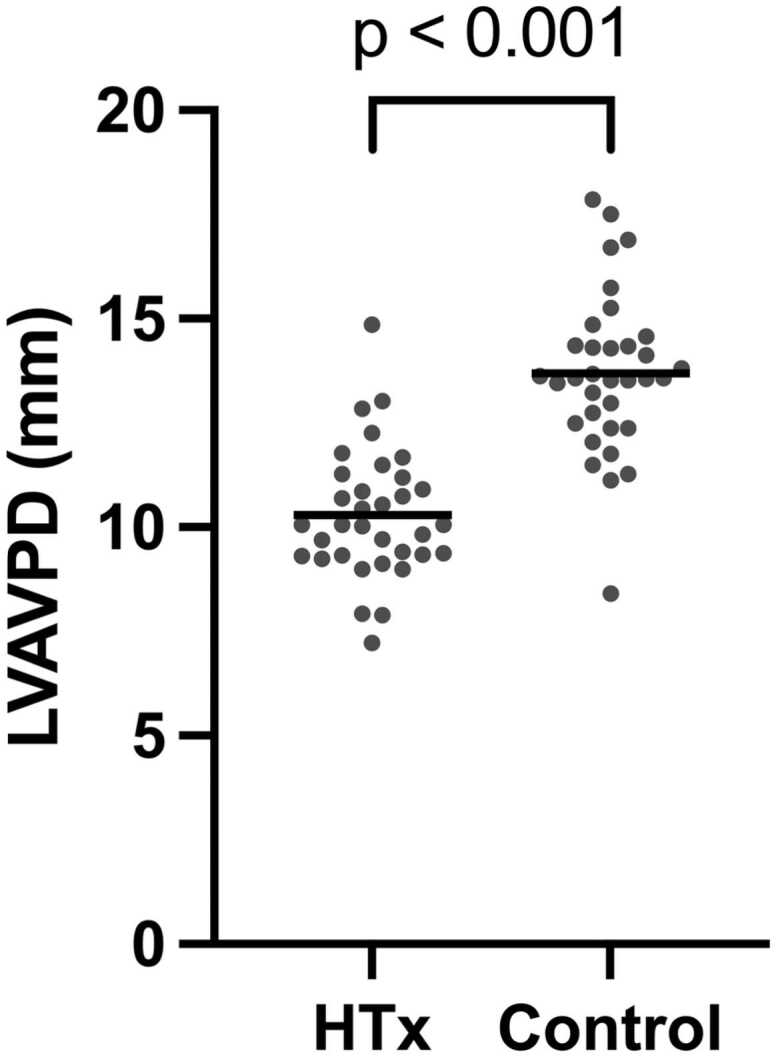
*Left ventricular atrioventricular plane displacement.* Left ventricular atrioventricular plane displacement was lower in transplanted patients compared to healthy controls.

**Figure 3:**
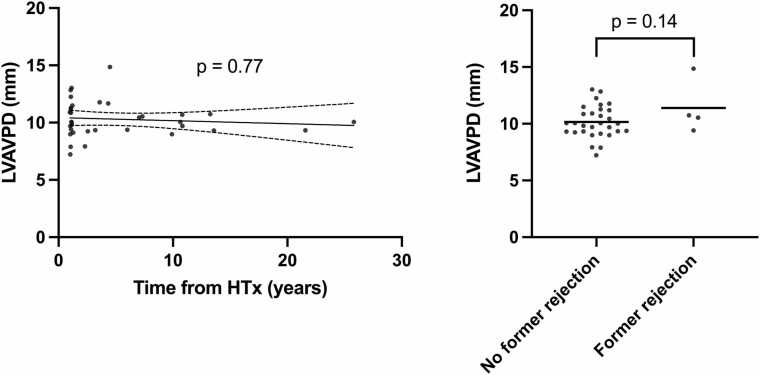
*Left ventricular atrioventricular plane displacement correlation with time from transplant **(a)** and previous rejections **(b)**.* There was no association with time from transplant or previous rejections by unpaired *t*-test.

Left ventricular AVPD was lower in the transplanted group compared to healthy controls (Figure 2, Table 2). There was no association between LVAVPD and time from transplant (Spearman correlation 0.05, *p* = 0.77) (Figure 3a) or between LVAVPD and previous rejections (10.2 vs 11.4 mm, *p* = 0.14) (Figure 3b). There was no association between LVAVPD and cardiac output at rest (*R*^2^ = 0.04, *p* = 0.62) in the transplanted patients.

**Figure 4:**
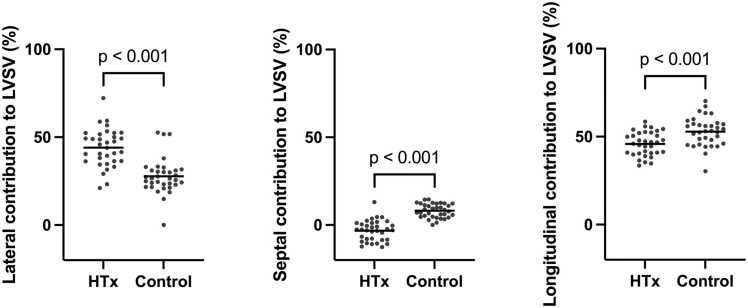
*Regional contribution to stroke volume.* Stroke volume was lower in the transplanted group even though an increased lateral contribution **(a)** in part compensated for the lower septal **(b)** and longitudinal **(c)** contributions.

As shown in Figure 4 and Table 2, longitudinal contribution to stroke volume was lower in the transplanted group when compared to healthy controls. Twenty-four patients (67%) in the transplanted group had a negative septal contribution to left ventricular stroke volume, in comparison with none in the control group. Furthermore, lateral contribution to stroke volume was higher in transplanted patients.

While there was no difference in LVEF between transplanted patients and healthy controls, stroke volume was lower in the transplanted patients. Cardiac output was higher in the transplanted patients due to higher HR.

Univariate linear regression analysis showed that transplant status, age, LVEDV, HR, and SV were correlated with LVAVPD (Table 3). In the multiple linear regression analysis, only transplant status, stroke volume, and age remained significantly associated with LVAVPD (Table 4).

**Table 3:** Univariate Linear Regression

Analyzed variable	Univariate linear regression analysis			
	β	95% CI	*R*^2^	*p*-value
HT status	−3.40	−4.23-(−2.55)	0.49	<0.01
Sex	0.58	−0.68-1.84	0.01	0.36
Age	−0.02	−0.08-(−0.00)	0.06	0.04
BSA	−0.46	−3.63-2.70	0.00	0.77
LVEDV	0.02	0.00-0.04	0.08	0.02
HR	−0.09	−0.13-(−0.06)	0.26	<0.01
SBP	0.00	−0.04-0.05	0.00	0.90
SV	0.79	1.58-4.75	0.19	<0.01

Univariate linear regression model of association with left ventricular atrioventricular plane displacement.

Abbreviations: BSA, body surface area; HR, heart rate; HT, heart transplant; LVEDV, left ventricularend-diastolic volume; SBP, systolic blood pressure; SV, stroke volume.

**Table 4:** Multivariate Linear Regression

Analyzed variable	Multivariate Linear Regression Analysis			
	*β*	95% CI	*R*^2^	*p*-value
HT status	2.94	1.93-3.94	0.44	<0.01
Age	−0.04	−0.07-(−0.01)	0.01	<0.01
LVEDV	−0.02	−0.05-0.00	0.71	0.07
HR	−0.00	−0.04-0.04	0.50	0.96
SV	0.07	0.02-0.11	0.73	0.03
HT-status, Age, LVEDV, HR, and SV combined	10.10	5.06-15.13	0.62	<0.01

Multivariate linear regression model of association with left ventricular atrioventricular plane displacement.

Abbreviations: HR, Heart rate; HT, heart transplant; LVEDV, Left ventricular end-diastolic volume; SV, Stroke volume.

### RBBB

There was no difference in stroke volume, LVAVPD, longitudinal, septal, or lateral contribution to stroke volume between transplanted patients with complete RBBB and those without complete bundle branch block (Table 5).

**Table 5:** Right Bundle Branch Block and Regional Left Ventricular Function

Analyzed variable	RBBB	No RBBB	*P*-value
Complete RBBB	11 (32%)	23 (68%)	
LVSV (ml)	81 (73-89)	94 (85-102)	0.05
LVAVPD (mm)	10.4 (9.5-11.3)	10.2 (9.5-11.0)	0.81
Longitudinal contribution to LVSV (%)	49 (46-53)	44 (41-47)	0.05
Septal contribution to LVSV (%)	−5 (−8-(−2))	−3 (−5-0)	0.36
Lateral contribution to LVSV (%)	47 (38-56)	43 (39-47)	0.27

Categorical data are presented as number with percentage, and continuous data are presented as mean with 95% CI.

Difference between means analyzed by unpaired *T*-test.

Significance level set to *p* < 0.05.

Abbreviations: LVAVPD, left ventricular atrioventricular plane displacement; LVSV, left ventricular stroke volume; RBBB, right bundle branch block.

### CPET

The median time between CMR and CPET was 2 (IQR 2-3) days in the heart-transplanted group. For six patients, the time between investigations was more than 3 days. Two patients had waited 36 and 146 days between CMR and the following CPET due to a back and extremity injury, respectively. Mean peak oxygen uptake was 18.8 ml x kg^−1^ x min^−1^. RER at maximal work was >1.10 for all but two patients, who both had an RER value of 1.09. There was no association between LVAVPD at rest and peak oxygen uptake in the transplanted group (*R*^2^ = 0.05, *p* = 0.20).

## Discussion

### Main findings

This study has shown that longitudinal heart function, as measured by LVAVPD and longitudinal contribution to stroke volume, is lower in heart-transplanted patients compared to healthy controls. However, this was in part compensated by an increased lateral contribution to stroke volume to maintain cardiac output.

Whilst the longitudinal, as well as the septal motion, was decreased at rest, the lateral contribution to stroke volume was increased in the heart-transplanted patients. A previous study in patients with heart failure and left bundle branch block scheduled for cardiac resynchronization therapy has shown similar results regarding regional function and lateral compensation for a decreased longitudinal and septal function, but these patients had both decreased systolic ventricular function and left bundle branch block, which was not the case in our cohort.^15^

### The effect of decreased LVAVPD on cardiac output and peak oxygen uptake

LVEF at rest was within normal values, but as previously described in heart transplant patients^16^ stroke volumes were decreased. The decrease in stroke volume seems to be compensated by HR. This association has previously been described as a result of allograft denervation,^17^, ^18^ and explains the slightly increased cardiac output at rest.

Peak oxygen consumption in the heart-transplanted patients was in line with previous reports.^19^, ^20^, ^21^ In contrast to what was seen in a previous study of 25 non-transplanted individuals with variation in age and physical form,^1^ we found no association between LVAVPD and peak oxygen uptake. A decreased ability among heart-transplanted patients to further recruit regional ventricular function during exercise could be suspected, and future investigations on longitudinal and regional pumping during exercise would be of value.

### Possible explanations of the altered regional function

Whether the altered mechanics are due to surgical alterations, denervation, tissue characteristics such as fibrosis from previous damage or ongoing subclinical rejection, electromechanical properties, interaction with the surrounding tissues, effects from immunosuppressant drugs, or a combination of several factors remains uncertain. Diastolic filling has previously been shown to be impaired and associated with the altered geometrical relationship between the left atrium and ventricle in transplanted patients,^22^ and it is possible that such surgical factors affect longitudinal function as well.

Decreased regional wall motion in transplanted hearts has been found prognostic for adverse outcomes and associated with CAV.^23^ In the current study, although limited by size, there was no association between CAV and longitudinal movement.

**Figure 5:**
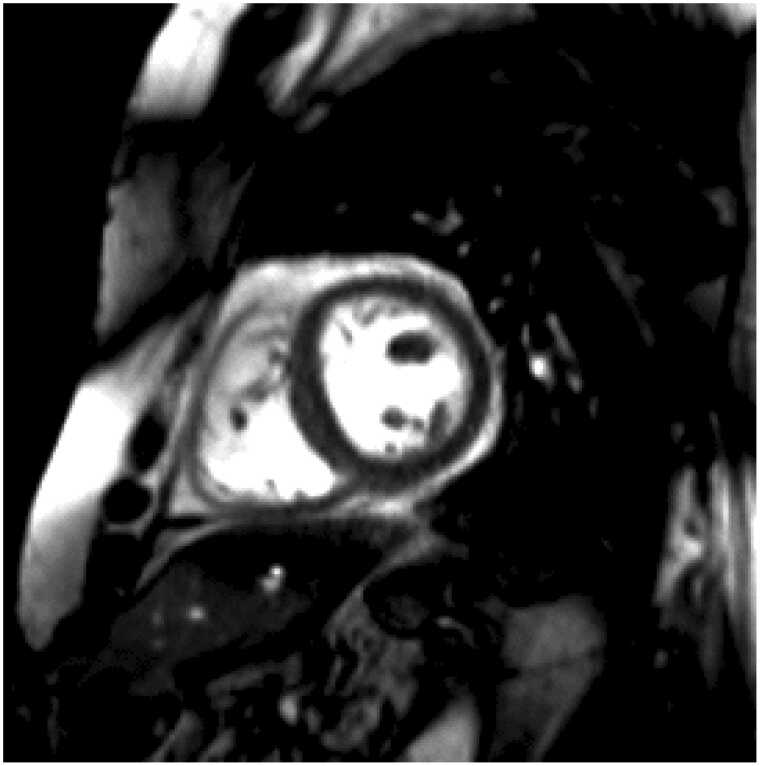
*(GIF): Paradox septal movement.* Mid-ventricular short axis view of the heart transplanted patient with the largest negative septal contribution to stroke volume. Note that despite septal myocardial thickening during systole, the net systolic movement of the septum is toward the right.

A negative contribution of septal motion to stroke volume was observed in the transplanted patients in the current study (Figure 5). Paradoxical septal motion is a phenomenon characterized by movement of the interventricular septum towards the right ventricle in systole, with normal muscular thickening. It is common after cardiac surgery, especially following valve replacement,^24^ and has previously been associated with reduced atrioventricular movement of the right ventricle.^25^ Buckberg and colleagues suggested that this is explained by the longitudinal motion of the right ventricle mainly being produced by septal fibers.^26^ Whether paradoxical septal motion is common in heart-transplanted patients, or whether the mechanism could cause decreased left ventricular longitudinal function in other groups, has not been previously described.

### Possible association with RBBB

There was no association between RBBB and LVAVPD or longitudinal contribution to left ventricular stroke volume in our material. RBBB is common in heart transplant recipients. New-onset RBBB within the first month of transplantation has previously been associated with both reduced biventricular function and worse long-term outcomes.^27^ Conduction abnormalities affect regional contraction,^28^, ^29^ and hence affect pump function. Post-operative RBBB in non-transplanted patients affect septal movement.^30^ As the investigated cohort is limited in size, we believe that the trends associated with RBBB should be interpreted with caution and tested in larger cohorts with power to determine the role of RBBB for pumping mechanics in heart transplant patients.

### The clinical significance of longitudinal pumping in heart failure

Longitudinal heart function measured with different methods has previously been shown to predict outcomes in different patient populations. As LVAVPD is measured as the perpendicular distance between the AV-plane at end diastole and end systole, it is a measure of pure longitudinal function.^12^ This can be compared to MAPSE, which is numerically close but also includes the effects of radial movement. In a previous study on 71 patients with pulmonary arterial hypertension, LVAVPD was shown to be a predictor of death and lung transplantation (HR 2.1, *p* = 0.02).^2^ In another cohort of 287 patients with heart failure with reduced ejection fraction, 1 mm decrease in LVAVPD was associated with a significant increase in 5-year cardiovascular mortality (HR 1.3, *p* < 0.001).^3^ In line with this, further emphasizing the importance of longitudinal function, MAPSE has been shown to exceed global longitudinal strain and LVEF in the prediction of death and hospitalization for heart failure.^31^ Equally, long axis strain assessed by CMR has been shown superior to LVEF in predicting future cardiovascular events and heart failure,^32^ to have a strong association with major adverse cardiovascular events in post myocardial infarction patients,^33^ and to predict cardiac adverse events in patients with non-ischemic dilated cardiomyopathy.^34^ In heart transplant recipients, tissue mitral annular displacement measured by two-dimensional speckle tracking echocardiography has recently been shown to be associated with survival.^35^ Reference values for the different measurements of longitudinal function in heart transplant recipients, and any potential association between LVAVPD and outcome, remains to be investigated.

### The clinical use of CMR and longitudinal function after heart transplant

The manuscript is based on data from our routine clinical post-transplant follow-up program. Due to availability and cost, most centers do not use CMR for standard surveillance, but its usefulness in diagnosing and excluding acute rejection is well established^36^, ^37^, ^38^, ^39^ and has even been shown cost-effective compared to standard care with repeated endomyocardial biopsies.^40^ In addition, CMR opens for assessment of myocardial perfusion, which may have potential to be used in CAV-surveillance.^41^ CMR carries the advantages of being non-invasive and without need of ionizing radiation or iodine contrast, which could potentially affect kidney function, and it is our belief that it will probably have a greater role in standard post-transplant follow-up in the near future.

The most common way to assess allograft dysfunction is echocardiographic measurement of ejection fraction. Our study shows that decreased longitudinal function may be present even with preserved LVEF. If our findings regarding LVAVPD may be translatable to other measures of longitudinal function, such as MAPSE measured by echocardiography, is presently uncertain. In addition, any association between rejection or clinical outcome and decreased LVAVPD in heart transplant patients remains to be investigated.

### Limitations

The main limitation of the findings is the small sample size of this single-center study. CPET investigations of our control group would also have added value.

We found no association between LVAVPD and exercise capacity, which may be explained by CMR investigations being performed at rest. Evaluation of longitudinal pumping during exercise, by CMR or echocardiography, could give valuable input in the future. The study was performed on available retrospective data, and no power calculation was performed for the main study nor the subanalysis on the potential association between RBBB and longitudinal function. For RBBB and longitudinal function, no association between the two was identified, but the limited number of patients in each group opens a risk of type II statistical error. We acknowledge the possibility that larger cohorts might have detected subtle differences.

The time gap between CMR and CPET in the transplanted cohort was somewhat varied, in one patient up to 146 days. No clinical suspicion of rejection or altered clinical status was present, but it cannot be excluded that the exercise capacity would have differed if the investigations had been carried out with more proximity.

It is uncertain if the results are unique for heart-transplanted patients or partially or completely a result of the surgery per se. It would be of value to compare the results to patients who have undergone thoracic surgery for other reasons than heart transplantation, preferably post valve replacement, as this is associated with paradoxical septal motion not confounded by regional variations in function that probably could confound an analysis of ischemic patients.

## Conclusion

While left ventricular longitudinal function was decreased in a cohort of heart-transplanted patients, stroke volumes were partially compensated by increased radial contribution from the lateral wall. There was no difference in longitudinal function between transplanted patients with and without a RBBB. In contrast to what is found in healthy controls, there was no significant association between longitudinal function and cardiac output at rest or with exercise capacity. Whether the mechanism of pumping is altered during exercise, or if there is an association between left ventricular longitudinal function and outcome in heart transplant recipients, remains to be investigated.

## CRediT authorship contribution statement

Conceptualization: Grunde Gjesdal, Oscar Ö Braun, Henrik Engblom, Håkan Arheden, and Katarina Steding-Ehrenborg. Methodology: Grunde Gjesdal and Katarina Steding-Ehrenborg. Formal analysis: Grunde Gjesdal, Anna Székely, and Katarina Steding-Ehrenborg. Writing – original draft: Grunde Gjesdal. Writing – review and editing: Grunde Gjesdal, Anna Székely, Oscar Ö Braun, Henrik Engblom, Håkan Arheden, and Katarina Steding-Ehrenborg.

## Disclosure statement

The authors declare that they have no known competing financial interests or personal relationships that could have appeared to influence the work reported in this paper. No relevant financial conflicts of interest.
